# Analgesia and patient comfort after enhanced recovery after surgery in uvulopalatopharyngoplasty: a randomised controlled pilot study

**DOI:** 10.1186/s12871-021-01458-8

**Published:** 2021-10-02

**Authors:** Fei Huang, Minxue Wang, Huixin Chen, Nan Cheng, Yanling Wang, Di Wu, Shaoli Zhou

**Affiliations:** Department of Anesthesiology, The Third Affiliated Hospital of Sun Yat-sen University, No. 600 Tianhe Road, Guangzhou, 510630 Guangdong Province China

**Keywords:** Uvulopalatopharyngoplasty, Obstructive sleep apnoea hypoventilation syndrome, Enhanced recovery after surgery, Post-operative pharyngeal pain, Patient comfort

## Abstract

**Background:**

Uvulopalatopharyngoplasty(UPPP) is the most prevalent surgical treatment of obstructive sleep apnea, but postoperative pharyngeal pain may affect patient comfort. The enhanced recovery after surgery pathway has been proved beneficial to many types of surgery but not to UPPP yet. The aim of this pilot study was to preliminarily standrize an enhanced recovery after surgery protocol for UPPP, to assess whether it has positive effects on reducing postoperative pharyngeal pain and improving patient comfort, and to test its feasibility for an international multicentre study.

**Methods:**

This randomised controlled study analysed 116 patients with obstructive sleep apnoea (OSA) who were undergoing UPPP in a single tertiary care hospital. They were randomly divided according to treatment: the ERAS group (those who received ERAS treatment) and the control group (those who received traditional treatment). Ninety-five patients completed the assessment (ERAS group, 59 patients; control group, 36 patients). Pharyngeal pain and patient comfort were evaluated using a visual analogue scale (VAS) at 30 min and at 6, 12, 24 and 48 h after UPPP. Complications, hospitalisation duration, and hospital cost were recorded.

**Results:**

The VAS scores for resting pain and swallowing pain were significantly lower in the ERAS group than those in the control group at 30 min and at 6, 12, 24 and 48 h after surgery. Patient comfort was improved in the ERAS group. The hospitalisation duration and cost were comparable between the groups. The incidence of complications showed an increasing trend in the ERAS group.

**Conclusion:**

The ERAS protocol significantly relieved pharyngeal pain after UPPP and improved comfort in patients with OSA, which showed the prospect for an larger study. Meanwhile a potential increase of post-operative complications in the ERAS group should be noticed.

**Trial registration:**

Chinese Clinical Trial Registry (23/09/2018, ChiCTR1800018537)

## Background

Obstructive sleep apnoea (OSA) is defined as a partial or complete upper respiratory obstruction while sleeping and is closely related to hypertension, coronary artery disease, heart failure, neurocognitive dysfunction, depression and other complications [1–5]. The primary surgical treatment for OSA is uvulopalatopharyngoplasty (UPPP), which relieves the obstruction by removing or shortening the uvula and soft palate, sometimes together with tonsillectomy. However, UPPP carries a high anaesthesia-related risk, with a series of possible post-operative complications [6, 7]. Moreover, post-operative pain caused by deficient analgesia affects patient swallowing, food intake and movement, with negative impact on post-operative comfort and quality of life, leading to delayed recovery [8, 9]. In addition, the improper use of sedatives and analgesics can increase the risk of respiratory depression, bleeding and other adverse events [10, 11]. Therefore, it is necessary to improve perioperative management to decrease post-operative complications, reduce uncomfortable symptoms and enhance quality of life.

Enhanced recovery after surgery (ERAS) is a type of perioperative management that combines evidence-based approaches to relieve operative stress reactions and to protect from organ impairment, leading to better prognosis [12, 13]. The elements of ERAS include pre-operative optimisation of co-morbid conditions, avoidance of prolonged pre-operative fasting, routine anti-emetic prophylaxis, use of opioid-sparing analgesic techniques, maintenance of normothermia, post-operative early oral intake and early ambulation [14, 15]. In particular, adequate analgesia is an important part and a major pre-condition of ERAS [16, 17]. Pain, a significant factor leading to stress response, results in anxiety, delayed out-of-bed activity, poor wound healing and depressed intestinal peristalsis, thus affecting post-operative recovery, hospital discharge, quality of life and comfort level. Within the ERAS protocol, multimodal analgesia is considered as one of the most important components [18]. Multimodal analgesia is defined as the administration of several analgesic drugs and/or methods, acting through different mechanisms, to obtain better analgesic effect and fewer adverse effects, thus optimising the analgesic/adverse effect ratio [19]. The combination of different analgesic drugs and methods not only achieves a satisfactory analgesic effect, but also reduces the side effects of opioids and enhances the recovery from operation.

The ERAS protocol has been applied in colorectal surgery, hepatic surgery, gynaecology, arthroplasty and other contexts [12, 20–23] and was shown to significantly shorten hospitalisation duration and reduce post-operative complications, mortality and medical costs. However, there are few studies on its application in UPPP. The main purpose of this prospective pilot research is to draw up an ERAS protocol for UPPP, and to study the its positive effects on postoperative pharyngeal pain and patient comfort, assessing its feasibility for an international multicentre study.

## Methods

The aim of this prospective study was to investigate the impacts of the ERAS protocol based on multimodal analgesia on the prognosis of UPPP. This study was designed as a randomized controlled study in accordance with the Consolidated Standards of Reporting Trials guidelines, and performed in a single tertiary care hospital, the Third Affiliated Hospital of Sun Yat-sen University. It was registered at Chinese Clinical Trial Registry (23/09/2018, ChiCTR1800018537).

### Patients

According to the international classification of sleep disorders approved by the America Academy of Sleep Medicine, OSA was diagnosed through polysomnography in this study. The diagnosis of OSA can be confirmed if one of the following criteria is present:

- Five or more predominantly obstructive respiratory events (obstructive and mixed apnoeas, hypopneas or respiratory effort–related sleep arousals) per hour of sleep in a patient with one or more of the following: (1) sleepiness, non-restorative sleep, fatigue or insomnia symptoms; (2) waking up with breath-holding, gasping, or choking; (3) habitual snoring, breathing interruptions, or both, noted by a bed partner or another observer; (4) hypertension, mood disorder, congestive heart failure, atrial fibrillation, or type 2 diabetes mellitus.
- Fifteen or more predominantly obstructive respiratory events per hour of sleep regardless of the presence of associated symptoms or co-morbidities.

Patients with OSA, aged 18–65 years, undergoing elective UPPP were enrolled. All patients underwent polysomnography when admitted, and those with apnoea-hypopnoea index (AHI) between 5 and 14 per hour of sleep were classified as having mild OSA, between 15 and 30 per hour of sleep as moderate and > 30 per hour of sleep as severe. Exclusion criteria were (1) severe cardiac and/or pulmonary disorder; (2) severe diabetes; (3) contraindications related to the ERAS protocol; (4) inability to cooperate with evaluations; (5) other major surgeries in the last 6 months; (6) simultaneous participation in other clinical studies; and (7) refusal to participate. Cases with adverse events related to the ERAS protocol (local anaesthetic intoxication, severe anaphylaxis, gastrointestinal haemorrhage etc.) or post-operative complications (wound bleeding, infection, respiratory crisis, cardiovascular events, or death) were excluded from other analysis because of the disruption or incompleteness of either protocol, but they were included in the safety analysis.

The patients were randomly divided by a computer-generated simple randomisation schedule into the ERAS group or the control group (ratio: 1.5:1). This pilot study was a single-blind tral. The patients and the post-operative evaluator, but not the nurses, surgeons or anaesthesiologists, were blinded to the group allocation. Information about patient numbers and groups were concealed in an envelope kept by a staff member who did not participate in any step of the study process. The surgeons, the nurses, and the anesthetists were allowed to know the information in the envelopes perioperatively in order to provide corresponding treatments. The postoperative evaluation was carried out by a researcher who was blinded as well and did not take part in any other part of the study. The unblinding was finally carried out by a statistician. Patients who were blinded during the whole study, of different groups were not arranged in the same ward. General anaesthesia with nasotracheal intubation was performed on all patients.

### ERAS protocol

During the pre-operative period, patients were oriented about smoking and alcohol cessation and pulmonary function exercise and were given a loxoprofen sodium tablet (60 mg). Patients abstained for 8 h from solid food and for 2 h from fluids and were given 5 ml/kg of a carbohydrate drink 8 and 2 h before surgery. During the intra-operative period, patients received non-steroidal anti-inflammatory drugs (NSAIDs; flurbiprofen axetil 50 mg or parecoxib 40 mg iv), dexmedetomidine (0.5 μg/kg by iv pump) and a lidocaine-phenylephrine mixture for nasal dripping before induction. Dexamethasone (8 mg iv) was given after anaesthetic induction. Ropivacaine (0.3%) was injected into the arcus pharyngopalatinus and the superior and middle borders of the palatine tonsil by the surgeon for local anaesthesia prior to mucosa incision. Fentanyl (2–4 μg/kg iv) was given in the induction period, and remifentanil (0.05–0.1 μg/kg/min iv) was given for intra-operative analgesia. Oxycodone (0.05 mg/kg iv) combined with NSAIDs (flurbiprofen axetil 50 mg or parecoxib 40 mg iv) and tropisetron (5 mg iv) were administered post-operatively. The body temperature was monitored and kept above 36.0 °C during surgery. And restricted fluid therapy was adopted. The estimated blood loss volume was less than 30 ml; hence, no fluid preload was applied before surgery. Crystalloid fluid was given intraoperatively (1–3 ml/kg/h). To reduce bleeding, blood pressure was controlled at approximately 80% of the baseline. During the post-operative period, parecoxib 40 mg once a day and aerosol inhalation (normal saline) three times a day were administrated and ice water gargling four times a day was performed during hospitalisation. Patients were encouraged to engage in out-of-bed activities as soon as possible and to consume liquid food 2–4 h after surgery.

### Control group protocol

During the pre-operative period, information about the operation was given to patients as customary, but they were not given loxoprofen sodium tablet nor were they educated. Patients abstained from solid food and fluids for 8 h, and they were not given the carbohydrate drink before surgery. In the intra-operative period, fentanyl (2–4 μg/kg iv) was given in the induction period, and remifentanil (0.05–0.1 μg/kg/min iv) was given for intra-operative analgesia. Fentanyl (2–4 μg/kg iv) and tropisetron (5 mg iv) were administered post-operatively once. Body temperature, fluid volume and blood pressure were monitored and controlled as per common procedures. In the post-operative period, no NSAIDs were administered unless requested by the patient. The time to engage in out-of-bed activity was decided by the patients themselves, and food intake was recommended after the first flatus (Table 1).

**Table 1:** Implementation programs of the ERAS and control groups in the perioperative period

Period		ERAS Group	Control Group
Pre-operative phase	Education	Smoking and alcohol cessation and pulmonary function exercise	Traditional information was told
	Analgesia	Loxoprofen, NSAIDs	None
	Fasting	No solids for 8 h and no liquids for 2 h	No solids and liquids for 8 h
	Carbohydrate	8 h and 2 h before surgery	None
	Sedative	Dexmedetomidine before induction	None
Intraoperative phase	Analgesia	Fentanyl, remifentanil and oxycodone with NSAIDs	Fentanyl and remifentanil
	Local anaesthesia	For nasal mucosa and incision	None
	Temperature monitoring	>36.0 °C	Common processing
	Fluid therapy	Restricted fluid therapy	Common processing
	Controlled hypotension	Reducing MAP to 80% of the basic line	Common processing
	Anti-emetic prophylaxis	Dexamethasone 8 mg after induction and tropisetron 5 mg at the end of surgery	Only tropisetron 5 mg at the end of surgery
Postoperative phase	Analgesia	Parecoxib 40 mg QD	None
	Food-intake	2–4 h after surgery	None before the first flatus
	Out-of-bed activity	As soon as possible	Based on patients’ willingness

*ERAS* Enhanced recovery after surgery, *NSAID* Non-steroidal anti-inflammatory drug, *QD* Every day

### Data collection

The primary outcome was post-operative pharyngeal pain, measured by the visual analogue scale (VAS) scores related to resting pharyngeal pain and swallowing pain at 30 min and at 6, 12, 24 and 48 h after surgery.

The following data were collected and analysed: demographic and clinical data (including sex, age and body mass index), pre-operative complications, OSA severity, and American Society of Anesthesiologists, New York Heart Association and Mallampati classifications; intra-operative information (including the duration of surgery and anaesthesia, the volume of intra-operative intake and bleeding); data related to the permanence in the post-anaesthesia care unit (PACU) (including disorientation, sore throat, thirst, headache, chills and agitation); post-operative symptoms such as dizziness, nausea, vomiting and pruritus; VAS scores related to patient comfort level, the Riker sedation-agitation score, the Ramsay sedation score (at 30 min and at 6, 12, 24 and 48 h after surgery) and the water swallowing test score (at 6, 24 and 48 h after surgery); total length of stay, post-operative length of stay, total hospital cost and anaesthetic cost.

### Statistics

The primary outcome of this study is the VAS score of the postoperative pharyngeal pain after UPPP. The sample size was set based on the results of a preliminary experiment, where the VAS score of postoperative pharyngeal pain (6 h after UPPP) was 2.45(±2.37) in the ERAS group, and 4.26(±3.84) in the control group. For the power of 80% and α = 0.05, with the drop-out rate of 20%, the sample size was calculated as 69 in the ERAS group and 46 in the control group.

All data collected were analysed using IBM SPSS Statistics version 23 (IBM Corp., Armonk, NY, USA). Normally distributed data are described as mean ± standard deviation, and non-normally distributed ones are described as median [interquartile range; IQR]. Categorical data are described as frequency (proportion). Significance in the comparisons between the ERAS and control groups was assessed by the χ^2^ test for categorical variables and by the Student t-test (for normally distributed data) or the Mann–Whitney U test (for non-normally distributed data) for quantitative variables. *P* < 0.05 was considered statistically significant.

## Results

**Fig. 1:**
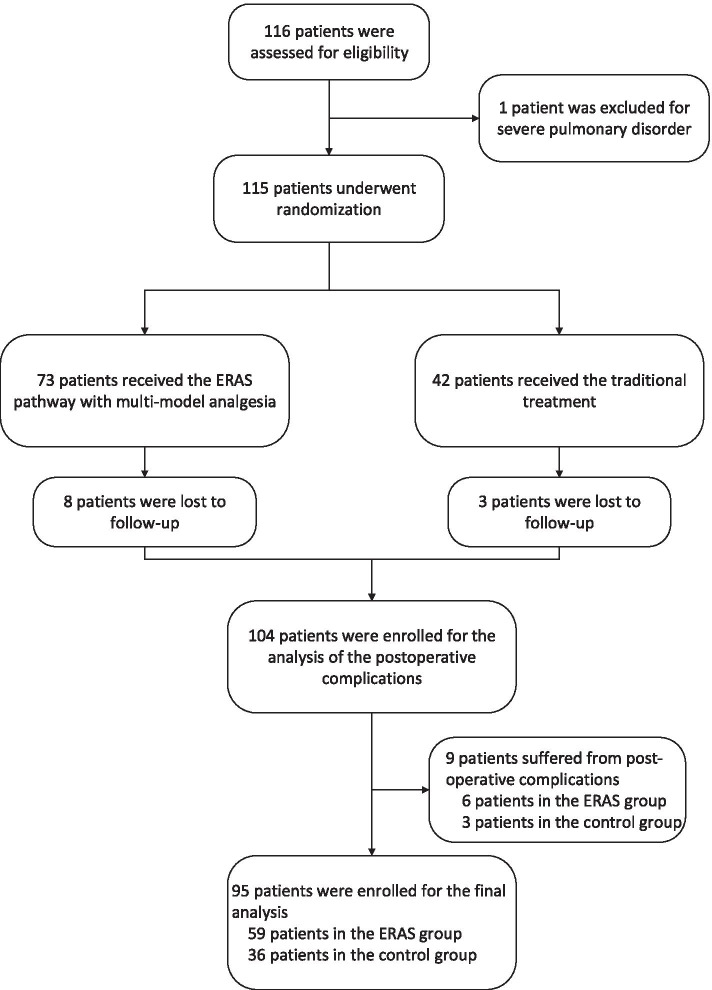
Study profile

Patient enrolment began on 25 October 2018. Among 116 enrolled patients, one patient was excluded for pre-operatively severe pulmonary disorder, and 95 completed the post-operative assessment (ERAS group: 59 patients; control group: 36 patients). Eleven patients were lost to follow-up, and 9 patients had serious post-operative complications (Fig. 1).

Demographic data and pre-operative conditions were comparable in the two groups (Table 2). Data in the intra-operative phase and the recovering period are reported in Table 3. There was no significant difference in the surgical duration between the two groups. The intraoperative dose of remifentanil was significantly lower in the ERAS group (306 ± 105 vs. 358 ± 91 μg; *P* = 0.016). Patients in the control group had more cases of disorientation, sore throat, thirst and agitation while recovering from anaesthesia than those in the ERAS group (*P* = 0.001, 0.022, 0.004 and 0.001, respectively).

**Table 2:** Demographic characteristics of the ERAS and control groups

Characteristic	ERAS Group(n = 59)	Control Group(n = 36)	***P*** value
**Age (years, mean ± SD)**	41.71 ± 11.72	40.56 ± 12.14	0.646
**Sex, male(n(%))**	48(81.36%)	32(88.89%)	0.329
**BMI (kg/m**^**2**^**, mean ± SD)**	26.75 ± 3.33	26.56 ± 3.11	0.783
**ASA classification(n(%))**			0.703
Class I	27(45.76%)	15(41.67%)	
Class II	29(49.15%)	19(52.78%)	
Class III	3(5.09%)	2(5.55%)	
**NYHA classification(n(%))**			0.927
Class I	49(83.05%)	30(83.33%)	
Class II	10(16.95%)	6(16.67%)	
**Mallampati classification(n(%))**			0.394
Class I	7(11.86%)	8(22.22%)	
Class II	28(47.46%)	14(38.89%)	
Class III	21(35.59%)	14(38.89%)	
Class IV	3(5.09%)	0(0%)	
**OSAHS severity(n(%))**			0.557
Mild	10(16.95%)	9(25.00%)	
Moderate	16(27.12%)	8(22.22%)	
Severe	33(55.93%)	19(52.78%)	
**Complications(n(%))**	15(25.42%)	10(27.78%)	0.800
Hypertension	15(25.42%)	9(25.00%)	0.963
Diabetes	2(3.39%)	2(5.56%)	0.610

*ERAS* Enhanced recovery after surgery, *SD* Standard deviation, *BMI* Body mass index, *ASA* American Society of Anesthesiologists, *NYHA* New York Heart Association, *OSAHS* Obstructive sleep apnea-hypopnea syndrome

**Table 3:** Information on OR and PACU of the ERAS and control groups

	ERAS Group (n = 59)	Control Group (n = 36)	***P*** value
Surgical duration (min, mean ± SD)	65.48 ± 16.32	63.94 ± 18.24	0.421
Intraoperative in-put (ml, median (IQR))	750.0(600.0–750.0)	750.0(750.0–837.5)	0.079
Intraoperative bleeding (ml, median (IQR))	15(10–20)	20(10–20)	0.411
Dose of remifentanil (μg, mean ± SD)	306 ± 105	358 ± 91	0.016
Situations in PACU(n(%))			
Disorientation	6(10.17%)	14(38.89%)	0.001
Sore throat	34(57.63%)	29(80.56%)	0.022
Thirst	14(23.73%)	19(52.78%)	0.004
Headache	0(0%)	1(2.78%)	0.198
Chill	1(1.69%)	1(2.78%)	0.721
Agitation	0(0%)	6(16.67%)	0.001

*OR* Operating room, *PACU* Post-anesthesia care unit, *ERAS* Enhanced recovery after surgery, *SD* Standard deviation, *IQR* Interquartile range

### Post-operative evaluation

**Fig. 2:**
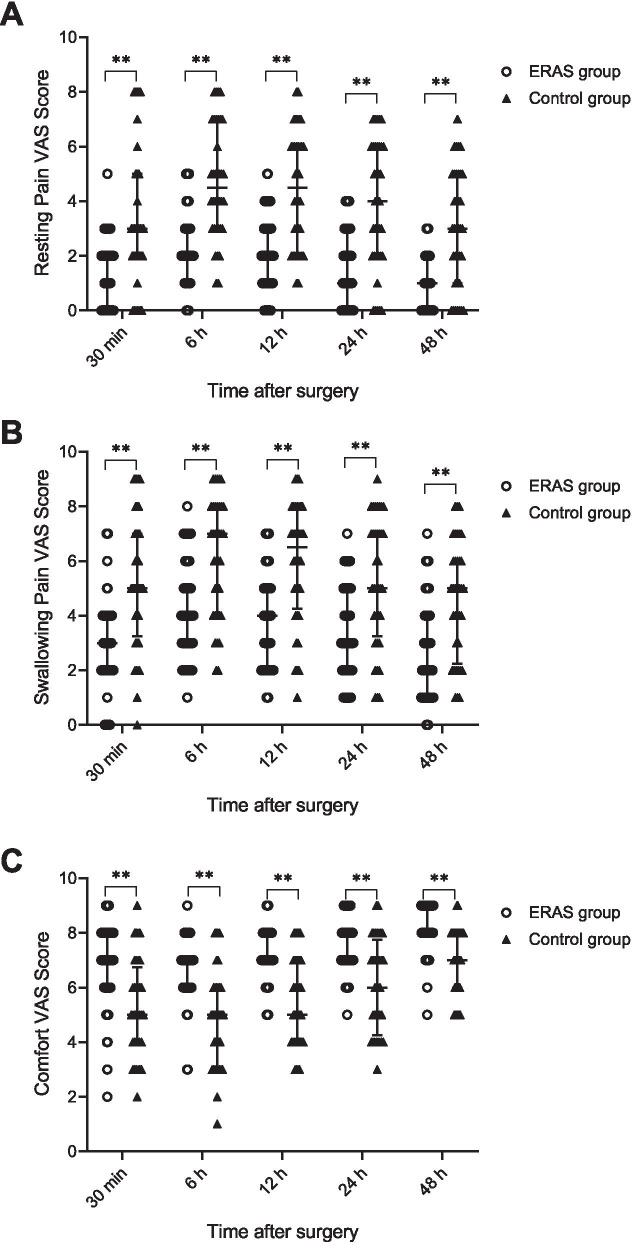
Visual analogue scales for post-operative pharyngeal pain and patients’ comfort. **P < 0.001

For both resting and swallowing throat pain at 30 min and at 6, 12, 24 and 48 h after surgery, the VAS scores of the ERAS group were lower than those of the control group (Fig. 2). In agreement with a previous study [24], the most painful period was within the first 12 h after surgery. The median VAS scores for swallowing pain were 4 [IQR: 3–5] in the ERAS group and 7 [IQR: 4–8] in the control group (*P* < 0.001). Patients in the ERAS group felt more comfortable during the whole post-operative period (Table 4). However, other factors were comparable between the ERAS group and the control group, including dizziness, post-operative nausea and vomiting (PONV), pruritus, the Riker sedation-agitation score (except for 30 min after surgery, *P* < 0.001) and the Ramsay sedation score (Table 5). In addition, the ERAS group performed better in the water swallowing test 6 and 12 h after surgery (*P* = 0.009 and 0.017, respectively).

**Table 4:** The VAS score of postoperative pain and patient comfort of the ERAS and control groups

	ERAS Group (n = 59)	Control Group (n = 36)	***P*** value
**Resting pain VAS score (median(IQR))**			
30 min after surgery	2(0–2)	3(2–5)	<0.001
6 h after surgery	2(1–3)	4.5(3–7)	<0.001
12 h after surgery	2(1–3)	4.5(2–6)	<0.001
24 h after surgery	1(0–3)	4(2–6)	<0.001
48 h after surgery	1(0–2)	3(1–5)	<0.001
**Swallowing pain VAS score (median(IQR))**			
30 min after surgery	3(2–4)	5(3.25–7)	<0.001
6 h after surgery	4(3–5)	7(4–8)	<0.001
12 h after surgery	4(2–5)	6.5(4.25–8)	<0.001
24 h after surgery	3(2–5)	5(3.25–7)	<0.001
48 h after surgery	2(1–4)	5(2.25–6)	<0.001
**Patient comfort VAS score (median(IQR))**			
30 min after surgery	7(6–8)	5(4–6.75)	<0.001
6 h after surgery	7(6–7)	5(3–6)	<0.001
12 h after surgery	7(7–8)	5(4–7)	<0.001
24 h after surgery	8(7–8)	6(4.25–7.75)	<0.001
48 h after surgery	8(8–9)	7(6–8)	<0.001

*VAS* Visual analogue scale, *ERAS* Enhanced recovery after surgery, *IQR* Interquartile range

**Table 5:** Other postoperative evaluations of the ERAS and control groups

	ERAS Group (n = 59)	Control Group (n = 36)	***P*** value
**Postoperative symptoms(n(%))**			
Dizziness	8(13.56%)	10(27.78%)	0.086
Nausea and vomiting	1(1.69%)	4(11.11%)	0.046
Pruritus	5(8.47%)	3(8.33%)	0.981
**Riker sedation-agitation score (median(IQR))**			
30 min after surgery	4(4–4)	4(3–4)	<0.001
6 h after surgery	4(4–4)	4(4–4)	0.869
12 h after surgery	4(4–4)	4(4–4)	1.000
24 h after surgery	4(4–4)	4(4–4)	1.000
48 h after surgery	4(4–4)	4(4–4)	1.000
**Ramsay sedation score (median(IQR))**			
30 min after surgery	2(2–2)	2(2–2)	0.221
6 h after surgery	2(2–2)	2(2–2)	1.000
12 h after surgery	2(2–2)	2(2–2)	1.000
24 h after surgery	2(2–2)	2(2–2)	1.000
48 h after surgery	2(2–2)	2(2–2)	1.000
**Water swallow test score (median(IQR))**			
6 h after surgery	2(2–2)	2(2–4)	0.009
24 h after surgery	2(2–2)	2(2–4)	0.017
48 h after surgery	2(2–2)	2(2–2)	0.464

*ERAS* Enhanced recovery after surgery, *VAS* Visual analogue scale, *IQR* Interquartile range

### Hospital stay and cost

The total and post-operative length of stay did not differ significantly between the two groups (*P* = 0.284 and 0.340, respectively). Moreover, the total cost of hospitalisation and the anaesthetic cost were also comparable (*P* = 0.195 and 0.749, respectively; Table 6).

**Table 6:** Hospital stay and cost of the ERAS and control groups

	ERAS Group (n = 59)	Control Group (n = 36)	***P*** value
**TLOS (days, median(IQR))**	8(7–10)	8(7–9)	0.284
**PLOS (days, median(IQR))**	5(4–7)	4(4–5)	0.340
**Total hospitalisation cost (yuan, mean ± SD)**	19,478 ± 2570	18,543 ± 2033	0.195
**Anaesthetic cost (yuan, mean ± SD)**	4028 ± 416	3893 ± 411	0.749

*ERAS* Enhanced recovery after surgery, *TLOS* Total length of stay, *PLOS* Postoperative length of stay, *SD* Standard deviation

### Complications

Nine patients had complications (six in the ERAS group and three in the control group), including hypoxia (one in the ERAS group), gastrointestinal haemorrhage (one in the ERAS group), wound infection (one in the control group) and wound bleeding (four in the ERAS group and two in the control group), of which three cases occurred within 24 h after surgery (two in the ERAS group and one in the control group). The sub-analysis of complications is presented in Table 7.

**Table 7:** Complications of the ERAS and control groups

	ERAS Group (n = 65)	Control Group (n = 39)	***P*** value
**Total complication(n(%))**	6(9.23%)	3(7.69%)	0.787
**Wound bleeding(n(%))**	4(6.15%)	2(5.13%)	0.828
Primary bleeding	2(3.08%)	1(2.56%)	0.880
Secondary bleeding	2(3.08%)	1(2.56%)	0.880
**Gastrointestinal Haemorrhage(n(%))**	1(1.54%)	0(0%)	0.436
**Hypoxia(n(%))**	1(1.54%)	0(0%)	0.436
**Wound infection(n(%))**	0(0%)	1(2.56%)	0.195

*ERAS* Enhanced recovery after surgery

## Discussion

OSA is an increasingly prevalent health problem worldwide due to rising rates of obesity, longer life expectancy and more sensitive screening methods [25, 26]. Its correlation with cardiovascular diseases, metabolic diseases and depression has been recognised in most of the previous studies [1–5, 27, 28]. To this day, UPPP remains the most commonly performed surgery for OSA, although it is characterised by considerable post-operative complications and intensive pharyngeal pain [9, 29]. Poorly controlled post-operative pain not only reduces patient satisfaction but also has adverse consequences [30]. Furthermore, because of obesity and difficulty in airway management, post-operative analgesia for patients with OSA requires special caution [10, 11, 31]. In particular, opioid overdose is not rare in patients with OSA, and opioid-induced respiratory depression is more prevalent in these patients [32]. In this study, the ERAS protocol with multimodal analgesia applied to UPPP provided significant post-operative pain reduction and decreased the intraoperative dose of opioids.

The ERAS pathway has been developed for more than 25 years. It was initially applied to colorectal surgery, and it is now being applied to most major surgical specialties. Normalised ERAS protocols and evidence-based guidelines for specific surgeries have been updated by the ERAS Society, an international organization and an authority on ERAS, which was founded in 2001 (www.erassociety.org). In this study, compared with the control group whose treatments were based on traditional clinical works and the personal experiences of anesthetists, the standardized protocol in ERAS group was drawn up referring to evidence-based guidelines recommended by the ERAS Society. The core difference of ERAS group is that multimodal analgesia was taken to reduce the use of opioids, and thus the side effects. The common elements of the ERAS protocol, including optimising pre-operative conditions, avoiding prolonged fasting, carbohydrate loading before surgery to minimise insulin resistance, multimodal analgesia, anti-inflammatory drugs to reduce the inflammatory response, minimising fluid shifts, maintenance of normothermia, precaution of PONV, early oral intake and ambulation [15], were adopted for UPPP in this study. However, unlike other major surgical specialties, which are focused on length of hospital stay, the key point of UPPP is the management of post-operative pharyngeal pain. Thus, in this study we emphasised the multimodal analgesia in the ERAS protocol for UPPP.

Multimodal analgesia is the essential part of the ERAS protocol. In this study, we used a combination of NSAIDs and intra-operative ultrashort-acting opioids, which were also recommended by published guidelines [19], and local anaesthesia as perioperative multimodal analgesia. On the one hand, NSAIDs relieve pain by inhibiting the composition of prostaglandins and by preventing central or peripheral sensitization. A retrospective study [33] demonstrated that perioperative application of NSAIDs provided adequate analgesia and reduced opioid use. On the other hand, the effects of anti-inflammation reduce postoperative occurrence of tissue oedema, potentially improving patient comfort by alleviating swallowing pain and breathing difficulties. Compared with the control group, the ERAS protocol significantly relieved pharyngeal pain in both resting and swallowing conditions after UPPP (all *P* < 0.001). Xie et al. [24] showed that NSAIDs combined with local anaesthesia provides more adequate post-operative analgesia. However, their sample size was small (n = 40), and the comparison was performed only between local anaesthesia with and without NSAIDs. Ponstein et al. [34] reported 3 cases in which continuous lesser palatine nerve block was used for post-operative analgesia after UPPP. However, the technique of catheter placement was not mature, resulting in catheter migration and leakage. Therefore, instead of nerve block, we chose to combine local anaesthesia with NSAIDs in this study. In addition, the dexamethasone used in this study also served as an adjunctive medication of multimodal analgesia. Dexamethasone was proven to have positive effects on reduction of postoperative swelling [35] which leads to physical strain and increased pain. A meta-analysis [36] showed that a single dose of systemic dexamethasone (more than 0.1 mg/kg) used as adjunct was effective in relieving postoperative pain at rest and with movement. Additionally, lower doses (4–5 mg, i.v.) were enough for prevention of PONV [37], potentially avoiding acute change of wound tensions along with fierce pharyngeal pain. Our results showed that multimodal analgesia provides a significant improvement in post-operative pain scores, and it was more feasible. Patient comfort was significantly promoted in the ERAS group in this study as well, and the multimodal analgesia may have played an important role in such improvement. In addition, the Riker sedation-agitation score was improved in the ERAS group (median: 4 [IQR: 4–4] vs. median: 4 [IQR: 3–4], *P* < 0.001) 30 min after operation. Abdelmageed et al. [38] showed that adequate sedation decreases the duration of mechanical ventilation, PACU stay and bleeding, whereas Xu et al. [39] showed that dexmedetomidine provided effective sedation after UPPP. As a highly selective alpha2 agonist, dexmedetomidine has both mild analgesic and sedative effects. Perioperative application of dexmedetomidine evidently reduces the total required dose of anaesthetics and opioids [40]. Duan *et al.* [41] demonstrated in 2018 that perioperative dexmedetomidine is effective for improving postoperative delirium. In our study, the incidences of symptoms related to delirium were significantly lower in the ERAS group than in the control group (*p* values for both disorientation and agitation were lower than 0.001). Meanwhile, fewer respiratory depressant effects were observed in dexmedetomidine than in other sedatives [42], implying potential benefits on reduction of perioperative airway complications in patients with OSA. In this study, dexmedetomidine was used as a sedative to relieve patient anxiety before operation, as an adjunctive medication for controlled hypotension procedures, and as an adjunct for early postoperative analgesia and sedation, leading to lower pharyngeal pain VAS scores and improvement of Riker sedation-agitation scores in the ERAS group. Although there was potentially increased risk of intraoperative bradycardia and hypotension with dexmedetomidine and remifentanil [43], the blood pressure in the ERAS group was controlled by vasoactive agents. Therefore, no hemodynamic events were observed in this study. Patients with bradycardia ≤50 bpm were treated with atropine and conditions were recorded as needed. However, no bradycardia treatment was necessitated during this study.

Although there was no significant difference in the incidence of complications between the two groups in this study, the patients in the ERAS group were more likely to have complications than those in the control group (9.23% vs. 7.69%, *P* = 0.787). The most common complication was wound bleeding, classified as primary bleeding (within 24 h after surgery) and secondary bleeding (after 24 h) [44]. Although the incidence of total wound bleeding, primary bleeding, and secondary bleeding was comparable between the two groups (*P* = 0.828, 0.880 and 0.880, respectively), the data implied that the ERAS protocol led to an increase in post-operative haemorrhage. This trend was attributed to the use of NSAIDs. However, previous conclusions about the impact of NSAIDs were controversial [45, 46]. The safety of the ERAS protocol in UPPP should be further studied and verified in an international multicentre study. Nevertheless, patients in the ERAS group performed better in the water swallowing test 6 and 24 h after UPPP, suggesting that the ERAS protocol may benefit the recovery of the swallowing function and reduce the risk of aspiration. Meanwhile, the incidence of post-operative nausea and vomiting was also reduced in the ERAS group (*P* = 0.046). Furthermore, the PLOS and TLOS are expected to be shorter and the cost lower in the ERAS group according to most studies related to major surgery; however, in this study, there was no difference between the two groups. Since a shorter LOS or a lower cost is mostly attributed to the enhanced recovery of organs impacted by major surgery and general anaesthesia, this advantage of the ERAS protocol may not apply to UPPP, which is a relatively minor surgery. In fact, the techniques and medicines used in the ERAS group were more expensive, and the cost should be higher. Moreover, in the ERAS group, perioperative internal complications necessitating extra examinations and treatments were documented in several patients. However, as this study showed no difference in PLOS, TLOS, and medical costs between the two groups, it can be assumed that the patients in the ERAS group benefited from early out-of-bed activity and food intake, which potentially reduced the cost of medical care.

There were several limitations to this study. First, it was carried out in a single institution with a relatively unbalanced sample. We noticed before the experiment that the ERAS group performed significantly better than the control group. Therefore, ethical considerations led us to use a ratio of 1.5:1 for the randomisation. A multicentre randomised controlled study with a larger sample is needed to better assess the generalisability of our results on the ERAS pathway in UPPP. Second, we did not adopt the widely used Bruggrmann comfort scale, based on the pain evaluation that was repeated in this study, to assess patient comfort. We thus focused on the overall patient experience, and by following the theory behind the VAS, patients were allowed to evaluate their overall feelings at different levels. However, a more comprehensive and precise evaluation of patient comfort may be applied in future studies. Third, the follow-up was limited to 48 h after operation, whereas the post-operative pharyngeal pain of UPPP could persist for more than 72 h. Long-term follow-up and discharge regimens are expected in subsequent studies. Fourth, in particular, the assessment of post-operative pain based on VAS was completely subjective. Objective methods, such as measurement of serum concentrations of C-reactive protein and tumour necrosis factor α, should be considered in future investigations.

## Conclusion

In conclusion, the ERAS protocol based on multimodal analgesia significantly relieved pharyngeal pain and improved patient comfort after UPPP in patients with OSA. This pilot study implied a good prospect for the application of the ERAS protocol in UPPP, and showed its feasibility for a large scale study with the acceptable missing rate and good patient compliance. However, the limitations above in this pilot study should be completed, and the potential risk of the ERAS protocol should be noticed in the future.

## Data Availability

The datasets used and/or analysed during the current study are available from the corresponding author on reasonable request.

## Abbreviations

AHI: Apnoea-hypopnoea index
ERAS: Enhanced recovery after surgery
NSAID: Non-steroidal anti-inflammatory drug
OSA: Obstructive sleep apnoea
PACU: Post-anaesthesia care unit
PONV: Post-operative nausea and vomiting
UPPP: Uvulopalatopharyngoplasty
VAS: Visual analogue scale
